# Excitability-Independent Memory Allocation for Repeated Event

**DOI:** 10.3389/fnbeh.2022.860027

**Published:** 2022-04-27

**Authors:** Hye-Yeon Cho, Han-Sol Lee, Yire Jeong, Junho Han, Miran Yoo, Jin-Hee Han

**Affiliations:** ^1^Department of Biological Sciences, Korea Advanced Institute of Science and Technology, Daejeon, South Korea; ^2^KAIST Institute for the BioCentury, Korea Advanced Institute of Science and Technology, Daejeon, South Korea

**Keywords:** mice, fear conditioning, memory, engram, retraining, excitability, reorganization, lateral amygdala

## Abstract

How memory is organized in cell ensembles when an event is repeated is not well-understood. Recently, we found that retraining 24 h after the initial fear conditioning (FC) event induces turnover of neurons in the lateral amygdala (LA) that encodes fear memory. Excitability-dependent competition between eligible neurons has been suggested as a rule that governs memory allocation. However, it remains undetermined whether excitability is also involved in the allocation of a repeated event. By increasing excitability in a subset of neurons in the LA before FC, we confirmed that these neurons preferentially participated in encoding fear memory as previously reported. These neurons, however, became unnecessary for memory recall after retraining 24 h following initial FC. Consistently, the initial memory-encoding neurons became less likely to be reactivated during recall. This reorganization in cell ensembles, however, was not induced and memory was co-allocated when retraining occurred 6 h after the initial FC. In 24-h retraining condition, artificially increasing excitability right before retraining failed to drive memory co-allocation. These results suggest a distinct memory allocation mechanism for repeated events distantly separated in time.

## Introduction

Memory is represented by a specific subset of cells in the brain (Josselyn et al., 2015; Tonegawa et al., 2015). We refer to the population of cells that encode the memory of a particular event as the engram. Of the countless number of neurons, which neurons are selected to become the engram is governed by certain rules as addressed by the study of memory allocation. The prevailing studies on the engram and memory allocation have revealed that allocation of memory to neuronal population is governed by competition of neurons based on their excitability (Han et al., 2007, 2009; Zhou et al., 2009; Yiu et al., 2014; Rashid et al., 2016). Accordingly, neurons with higher level of excitability than their neighboring cells at the time of learning are preferentially recruited to encode memories. These findings have suggested a concept that memory allocation is a stochastic process rather than a pre-determined one.

Although a large number of the recent studies have greatly advanced our understanding of engram and memory allocation into distinct neuronal populations, which enables us to track and manipulate a specific memory engram, less is known about how memory of repeated event is organized into neuronal ensembles. Using auditory fear conditioning (FC) paradigm in mice, we recently found that amygdala engram cells turnover when FC event is repeated 24 h after initial FC (Cho et al., 2021). The initially recruited engram cells in the LA become unnecessary for memory recall after the repetition of FC, while the neurons active during retraining are engaged in encoding the memory. These findings suggest that memory is re-allocated into distinct cell ensembles with the repetition of FC event. It has been previously suggested that cellular excitability governs the allocation of two distinct events into cell populations in the LA and hippocampal CA1 area (Cai et al., 2016; Rashid et al., 2016). Artificially increasing excitability even enabled co-allocation of two distinct FC events occurring far apart in time that are normally separated into distinct cell populations (Rashid et al., 2016). Given these previous findings, we reasoned that if the same excitability rule is responsible for allocation of repeated events, increasing excitability of initially recruited engram cells at the time of retraining would allow memory to be kept in the same cell population.

In this study, we investigated this possibility by optogenetically manipulating an excitability of sparse neurons in the LA at the time of initial training and/or retraining. We again found that retraining resulted in the formation of a new memory trace that is less likely to overlap with the initially created memory engram, which supported a form of memory reorganization by turnover in the memory engram. Unlike for the case of two distinct events, however, we found that increasing excitability did not drive co-allocation into an overlapping cell population, which implies different memory allocation mechanisms for initial learning and relearning.

## Materials and Methods

### Mice

Adult hybrid (129/C57B/6) mice were group-housed (3–5 mice per cage) and maintained in 12-h light/dark cycle at a constant temperature of 22 ± 2°C and 40–60% humidity. Food and water were available *ad libitum* throughout the experiment.

### Virus Production

We used herpes simplex virus (HSV) vector for bidirectional manipulation of LA principal neurons. The HSV-hChR2-2A-eNpHR-2A-Venus vector was generated by inserting the hChR2-2A-eNpHR-2A-Venus fragment from the original vector (Tang et al., 2009), which was kindly provided by Dr. Rolf Sprengel, into the pHSVpuc vector. The expression of such opsins was driven by the constitutive IE4/5 promoter. We confirmed reliable cleavage of the two opsins and fluorescent reporter (Supplementary Figure 1) as reported before (Tang et al., 2009). HSV packaging was performed as previously described (Neve et al., 2005). Lipofectamine-based method was used to transfect host 2–2 cells with the HSV vector. Virus was packaged with the aid of replication-defective helper virus. This mixture was then passaged over 3 rounds of 2–2 cell transfection. The harvested virus was purified on a sucrose gradient, pelleted, and resuspended in 10% sucrose. The average virus titer was 7 × 10^7^ infectious units ml^−1^.

### Surgery

Mice were anesthetized with pentobarbital (83 mg kg^−1^ of body weight) by intraperitoneal injection. After mice were fully anesthetized, they were mounted and fixed on the stereotaxic frame. Small holes were drilled with an electrical driller at target LA sites on both hemispheres of the brain (AP −1.8 mm, ML ±3.5 mm, DV −4.3 mm). Virus was loaded in a glass pipette filled with water and 1.5 μl of mineral oil at the tip. An appropriate volume of virus (1.0 μl) was injected at a rate of 0.1 μl/min^−1^ at the targeted LA sites. The injection pipette was placed at the injection site for an additional 10 min to allow sufficient diffusion of the virus. After the injection electrode was slowly withdrawn, optic ferrules (200 μm core) were placed above the LA (AP −1.8 mm, ML ±3.5 mm, DV −3.8 mm) and fixed with dental cement, one side at a time. Mice were placed on the heating pad for recovery and returned to their home cages. We carefully performed surgery and monitored animals' general health. To help the recovery from the surgery, 1 ml of warm sterile saline was administered subcutaneously after the surgery. Sometimes, carprofen (5 mg/kg) was injected as an analgesic when necessary.

### Behavioral Procedures

Transgene expression with HSV amplicon vector (pHSVpuc) is known to be rapidly induced and short-lived (2–5 days) (Neve et al., 2005). Due to this transient nature of viral gene expression by HSV vector, we started behavior experiments 2 days after the viral infection. Then, 2 days after HSV virus injection and ferrule implantation surgery, mice were placed in the conditioning chamber (Coulbourn Instruments, PA, USA). They were allowed to freely explore the area for 2 min, after which 30 s of ChR2 light stimulation (473 nm, 20 Hz, 5 ms, 0.3 mW at fiber tip) was presented (ChR2 ON). Immediately following, tone (2.8 kHz, 90 dB, 30 s) was paired with a foot shock (0.5 mA, 2 s) by co-termination. After an additional 30 s in the chamber, mice were returned to their home cages. Then, 1 day later, mice were placed in context-shifted chamber with an acrylic floor and semicircular wall. After 2 min Pre-CS, conditioned tone was presented for the total of 2 min, 1st min of which was together with NpHR inhibition (561 nm, continuous, 3–5 mW at fiber tip; NpHR ON). NpHR light was turned off during the 2nd min (NpHR OFF) to observe freezing to tone without optogenetic silencing.

For retraining experiments, 6 or 24 h after the first conditioning, mice were conditioned with the same CS-US pairing protocol, but without the ChR2 pre-stimulation. The memory recall test with NpHR inhibition (same as above) was conducted the next day.

For retraining with excitability manipulation, ChR2 stimulation (same as above) was given immediately before the tone presentation of the re-conditioning session.

For Arc fluorescence *in situ* hybridization (FISH) experiment, the retrieval test consisted of 2 min Pre-CS followed by 1-min tone presentation. Mice were sacrificed 5 min after the initiation of the tone.

### *In vivo* Multi-Unit Recordings

*In vivo* multi-unit recordings were conducted as previously described (Jeong et al., 2021). Gold and PEDOT-TFP were coated on bare iridium electrode sites of an optic fiber-coupled 16-channel silicone probe (A1x16-poly2-5mm-50s-177-OZ16LP, Neuronexus) to lower impedance <200 kΩ. NanoZ (Multi Channel Systems) was used to produce direct current for electroplating (Charkhkar et al., 2016). For gold plating, both poles were flooded in 0.1 M NaClO_4_ containing 5 mM HAuCl_4_. A number of six brief pulses of DC (−0.1 μA, 2-s interval, 1-s pulse) were applied. For PEDOT-TFB, another solution (10 mM EDOT and 0.1 M tetrabutylammonium tetrafluoroborate in acetonitrile) was prepared and followed by 10 pulses of DC (+0.03 μA, 5-s interval, 1-s pulse). Before each recording session, impedance was measured in PBS to confirm that it was below 200 kΩ.

To validate the function of HSV-hChR2-2A-eNpHR-2A-Venus in the LA, HSV-injected mice were anesthetized with isoflurane (4% for induction and 1% for maintenance) and fixed in a stereotaxic frame. After the coated probe was placed into the LA, recording data were acquired *via* a PZ5 amplifier (Tucker-Davis Technologies) and a Zif-Clip adapter (ZCA32, Tucker-Davis Technologies). The amplified signal was processed using a TDT RZ5P processor and Synapse software (Tucker-Davis Technologies). The sampling rate was 25 kHz and band-pass filter ranged at 300–5,000 Hz was applied. Spikes were detected when amplitude was over than 5 standard deviations from the median. To obtain the raster plot, the timing of spiking was analyzed by custom-written MATLAB code. For drawing a histogram, multi-unit activities were binned at 0.5 Hz, with bins corresponding to different light conditions (no light, 473 nm light, 473 + 561 nm light). The lasers were controlled by TDT RZ5P processor and Synapse software with a custom-made waveform. The 5 ms of 473 nm laser was delivered at 20 Hz for 4 s, and the continuous 561 nm laser was delivered together for the last 2 s of 473 nm illumination. The light intensities were identical to that used for behavior experiments (0.3 mW for 473 nm and 3–5 mW for 561 nm).

### Immunohistochemistry

Immunostaining was conducted with HSV-injected brain to identify the cell type of neurons manipulated. Mice were perfused transcardially with 100 ml of phosphate-buffered solution (PBS) followed by the same volume of 4% paraformaldehyde (PFA). Brains were extracted and stored in 4% PFA overnight. Coronal brain sections (40 μm) were obtained using the vibratome (VT-1200S, Leica). Sections were washed in PBS and incubated with rabbit primary antibody against GFP (1:5,000, ab290, Abcam) and mouse primary antibody against calcium/calmodulin-dependent protein kinase IIα (CaMKIIα 1:1,000, 05-532, Millipore) at 4°C for 72 h. Sections were then incubated in Alexa Fluor 488-conjugated goat anti-rabbit (1:2,000, A-11008, Molecular Probes) and Alexa Fluor 594-conjugated goat anti-mouse (1:2,000, A-11005, Molecular Probes) secondary antibodies for 2 h at room temperature. Immunostained brain sections were mounted on gelatin-coated slides and counterstained with DAPI (4',6-diamidino-2-phenylindol) mounting medium (h-1200, Vector). Images were acquired using Zeiss LSM 780 upright confocal microscope.

### Histology

At the end of all behavioral experiments, animals were sacrificed and coronal brain sections were prepared as above. Brain sections were viewed under the fluorescence microscope (ECLIPSE 80i, Nikon) for histological verification of virus expression and ferrule placement. By reference to the Mouse Brain Atlas (Franklin and Paxinos, 2013), only the animals that showed restricted virus expression in the LA and ferrule tip location above the LA were selected and included for data analysis. Mice that were excluded showed off-target expression in surrounding areas (amygdalostrial transition area or central amygdala) or physical damage by the ferrule.

### Fluorescence *in situ* Hybridization

For FISH, mice were sacrificed 5 min after the tone retrieval tests. Their brain tissues were removed within 30 s and immediately placed in the isopentane solution contained in dry ice-ethanol slurry. The fresh-frozen brain was stored in −80°C deep-freezer until sectioning. For cryosection, 4–6 brain samples were placed in disposable paraffin embedding mold and filled and fixed with embedding media at −20°C. Brain samples were sectioned coronally in 20 μm thickness and thaw-mounted on SuperFrost glass slides (1255018, Fisher Scientific). Slides were stored at −80°C until proceeding with FISH. FISH was performed as previously described (Guzowski et al., 1999). Using DIG-conjugated anti-sense RNA probe corresponding to the full-length *arc* (~3 kb) and FITC-conjugated anti-sense probe against *venus* (~0.7 kb), *arc* and *venus* mRNA signals were detected in the LA. After hybridization with the riboprobes, DIG and FITC signals were detected with HRP-conjugated anti-DIG and anti-FITC antibodies, respectively. Further amplification and visualization of the target RNA signals were achieved by the incubation with tyramide signal amplification (TSA) kit. Arc was amplified with Cy5 TSA, followed by *venus* RNA signal amplification with FITC TSA. Sections were then counterstained with DAPI mounting medium. Images were acquired using LSM780 (KAIST Bio-Core Center) confocal microscope (Zeiss).

### Western Blot

To confirm the cleavage of 2A sites, we prepared two dishes of HEK293T cells on 100 mm culture dishes and infected one dish with 3 μl of HSV-hChR2-2A- eNpHR-2A-Venus. Then, 1 day after the infection, the cells were harvested and lysed in a 100-μl ice-cold lysis buffer (50 mM HEPES pH 8.0, 400 mM NaCl, 10% glycerol, 1% Triton X-100, 5 mM DTT) containing a protease inhibitor cocktail (11836153001, Roche). Total protein concentrations were measured by Bradford assay. Equal amounts of proteins (20 μg per lane) were separated on 7.5% sodium dodecyl sulfate-PAGE (SDS-PAGE) and transferred to polyvinylidene difluoride (PVDF) membrane using the Trans-Blot® Turbo™ Blotting System (Bio-Rad). After blocking with 5% non-fat dried milk (NFDM) in TNTX buffer (50 mM Tris-HCl, pH 7.5, 200 mM NaCl, 0.2% Triton X-100) for 30 min at room temperature, the membrane was incubated with primary antibody against Venus (ab290, Abcam, 1:10,000) in 3% bovine serum albumin (BSA) overnight at 4°C. Then, the membrane was incubated with HRP-conjugated goat anti-rabbit IgG secondary antibody (12–348, Millipore, 1:2,000) for 1 h at room temperature. The blots were developed using ECL solution (RPN2232, GE Healthcare) and detected with ChemiDoc MP imaging system (Bio-Rad).

### Cell Counting Analysis

Cell counting analysis was performed using IMARIS software in a blinded manner. For each animal, 3–5 sections were counted for nuclear *arc* and *venus* signals and divided by DAPI+ cells to obtain percentages. Only the sections that showed strong and specific *venus* signal in the LA region were selected for analysis. The resulting *arc*+ and *venus*+ percentages (per DAPI) were averaged over the analyzed sections for each animal. The proportion of initial engram cells reactivated during recall of single-trained or retrained memory was calculated as (*arc*+ and *venus*+ cell number) / (*venus*+ cell number). Same counting methods were used to compare ChR2 OFF and ChR2 ON groups in Supplementary Figure 4.

### Statistical Analysis

Prism 7.05 (GraphPad software) was used for all statistical analysis in this study. Two-way repeated measures ANOVA followed by *Sidak's* multiple comparison test was used to analyze freezing behaviors. The unpaired Student's *t*-test was used to analyze FISH cell counting results. All data were evaluated by a significance level of *p* < 0.05. Details of statistical analysis are presented in Supplementary Tables 1, 2.

## Results

### Fear Memory Is Allocated Into Optogenetically Excited Neurons in the LA

**Figure 1:**
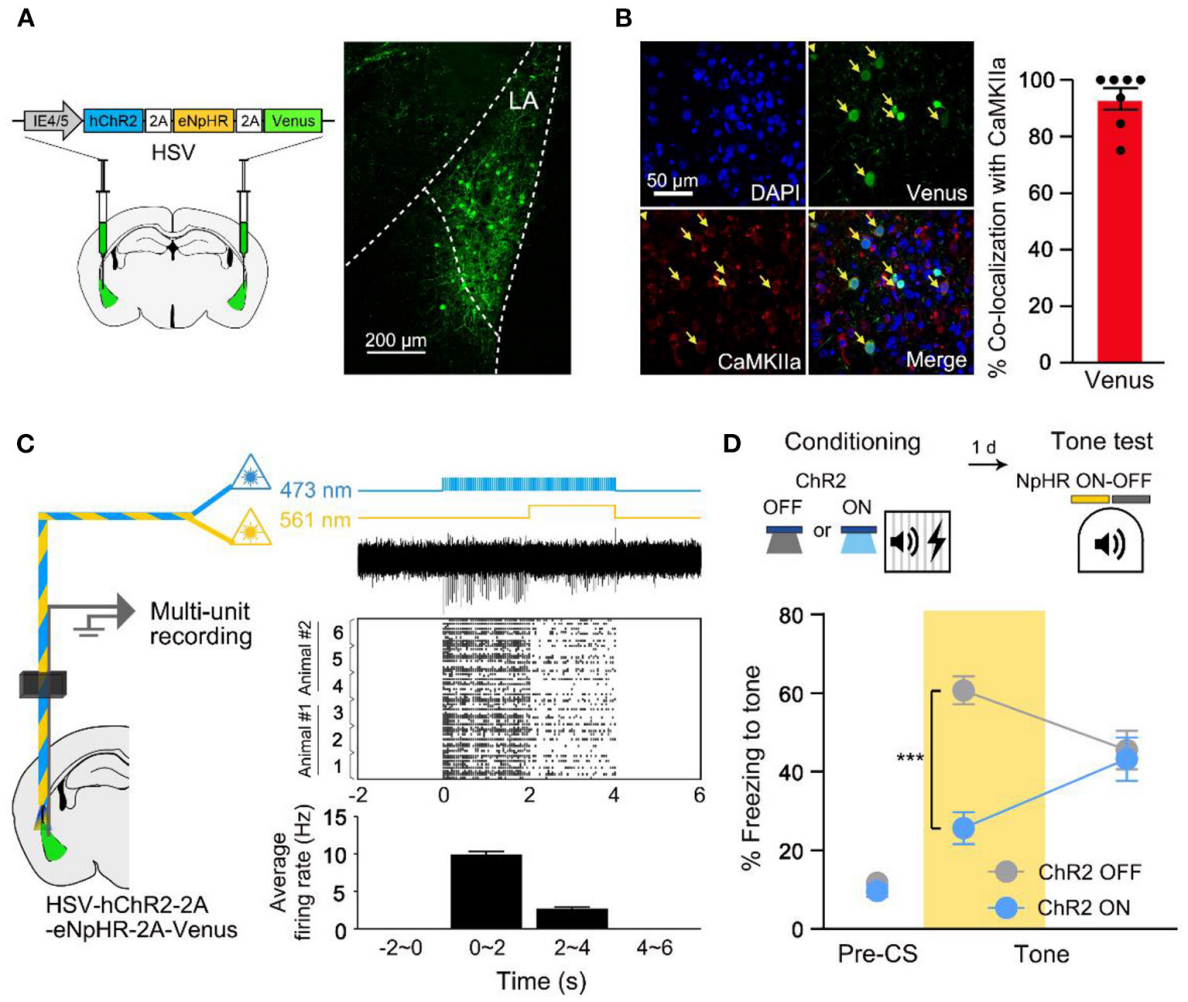
Fear memory is preferentially allocated to a subset of excitability manipulated LA neurons at the time of learning. **(A)** Schematic diagram of HSV injection in bilateral LA (left). Confocal microscopic image of virus expression in LA (right). **(B)** Representative confocal images showing most of the cells infected by the virus are CaMKIIα-positive (*n* = 7 mice). **(C)** Schematic diagram of *in vivo* multi-unit recording of HSV-expressing LA neurons under 473 and 561 nm laser illumination (left). Laser illumination protocols, representative multi-unit activity trace, raster plot of activities above threshold and histogram of average firing rate during 2 s time bin (3 independent trials from different sites in *n* = 2 mice; from top to bottom, right). **(D)** Behavior scheme for increasing excitability immediately before conditioning and inhibiting activity during tone memory recall (top). Freezing level during recall test with NpHR inhibition of excitability-manipulated (ChR2 ON; *n* = 13 mice) or random (ChR2 OFF; *n* = 10 mice) cells (bottom). Yellow shading indicates 561 nm light illumination hereafter. ***p < 0.001. Data are shown as mean ± SEM.

Memories are thought to be physically represented in the brain as distinct neuronal ensembles (Semon, 1921; Han et al., 2007; Liu et al., 2012; Josselyn et al., 2015; Tonegawa et al., 2015; Grewe et al., 2017). The prevailing studies identified sparse subset of neurons in the lateral amygdala (LA), especially those of increased excitability at the time of learning, as the auditory fear memory engram (Han et al., 2007, 2009; Zhou et al., 2009; Yiu et al., 2014; Rashid et al., 2016). In this study, we likewise biased memory allocation to a subset of LA neurons by optogenetically stimulating these cells immediately before training to track the fear memory engram. After artificial increase of excitability during auditory FC, we optogenetically silenced the same cells during conditioned tone retrieval, to test for memory allocation. For bidirectional regulation of LA neuron activity, we microinjected an HSV viral vector that encodes both channelrhodopsin-2 (hChR2) and halorhodopsin (eNpHR) in the LA (HSV-IE4/5-hChR2-2A-eNpHR-2A-Venus; Figure 1A). Using western blot analysis, we confirmed that ChR2, eNpHR opsins, and the Venus fluorescent protein were simultaneously expressed under the single promoter IE4/5 as previously reported (Supplementary Figure 1; Tang et al., 2009). Out of the HSV-infected neurons, approximately 98% were excitatory cells (Yiu et al., 2014) (CaMKIIα+; Figure 1B). We verified the bidirectional manipulation function using *in vivo* multi-unit optrode recordings in the LA of anesthetized mice injected with HSV-IE4/5-hChR2-2A-eNpHR-2A-Venus. The 473 nm blue light illumination (activating ChR2) induced an increase in firing rate in the LA neurons, which was, however, suppressed by simultaneously delivered 561-nm yellow light (activating NpHR3.0; Figure 1C) as reported previously (Tang et al., 2009; Gradinaru et al., 2010; Rashid et al., 2016). To manipulate the allocation of auditory fear memory into the HSV-infected neurons, ChR2 stimulation (20 Hz, 5 ms, 30 s) was given immediately before conditioning. Our auditory FC trial consisted of 30-s tone (conditioned stimulus, CS) that co-terminated with 2-s foot shock (unconditioned stimulus, US). To confirm memory allocation to the excited population, the same subset of neurons manipulated immediately before conditioning was inhibited during fear memory retrieval. Compared with a control group, NpHR inhibition reduced freezing only in the group that received excitability increase before conditioning (ChR2 ON; Figure 1D). Inhibiting a similar random population of LA neurons had no effect on memory recall (ChR2 OFF). Therefore, we verified memory allocation to more excitable neurons at the time of learning. The inhibitory effect was specific to impairing auditory fear memory, as the optogenetic silencing had no effect on retrieval of the context fear memory (Supplementary Figure 2).

### The Initially Recruited Engram Cells Become Unnecessary for Memory Recall After Retraining

**Figure 2:**
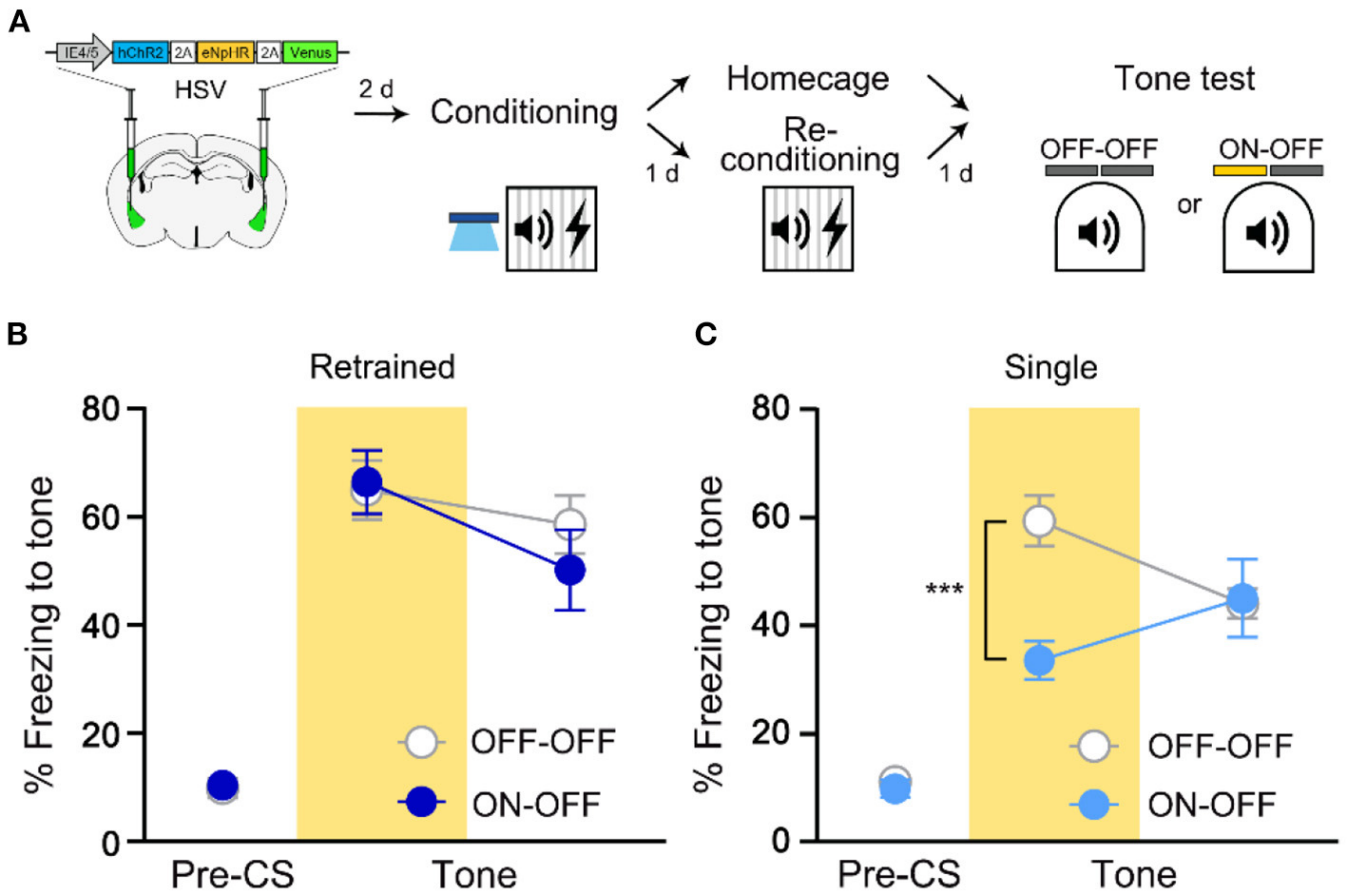
Recall of fear memory after retraining is not dependent on LA ensemble initially allocated to engram. **(A)** Schematic diagram of injection of HSV virus vector in the bilateral LA (left) and behavior experiment scheme (right). **(B)** Behavior results for tone recall test with (ON-OFF; *n* = 8 mice) or without (OFF-OFF; *n* = 7 mice) NpHR inhibition after re-conditioning. **(C)** Behavior results for tone recall test with (ON-OFF; *n* = 9 mice) or without (OFF-OFF; *n* = 9 mice) NpHR inhibition 2 days after training. ***p < 0.001. Data are shown as mean ± SEM.

To investigate whether the same cells initially recruited for memory allocation by optogenetic excitation remain necessary for memory recall even after retraining, we traced the memory-allocated cells after retraining conducted 1 day after the initial training (re-conditioning in Figure 2A). In this experiment, optogenetic excitation was used only during initial training but not during retraining. We repeated the FC protocol using the same conditioned and unconditioned stimuli during retraining. When tested 1 day after retraining, mice in NpHR ON group showed no significant difference in freezing level compared to that of NpHR OFF control (Figure 2B). Fear memory recall was not affected by the inhibition of the artificially allocated cells after re-conditioning. It is possible that HSV viral expression declined following the extended experimental timeline of the retraining group. However, the lack of NpHR inhibition effect on the freezing response was not simply due to delayed test day, as a single training group showed a significant decrease in freezing by 561 nm light even when tested at the same delayed time point (2 days after first training; Figures 2A,C). Thus, the initially manipulated ensemble was no longer necessary for recall of re-conditioned memory. This change was specific to retraining. If mice were exposed to CS alone, the NpHR inhibition during memory recall significantly impaired freezing (Supplementary Figure 3). The presence of the learning stimulus was thus important for retrained memory to become independent of the initial engram.

### Initial Engram Cells Become Less Likely Reactivated During Memory Recall After Retraining

**Figure 3:**
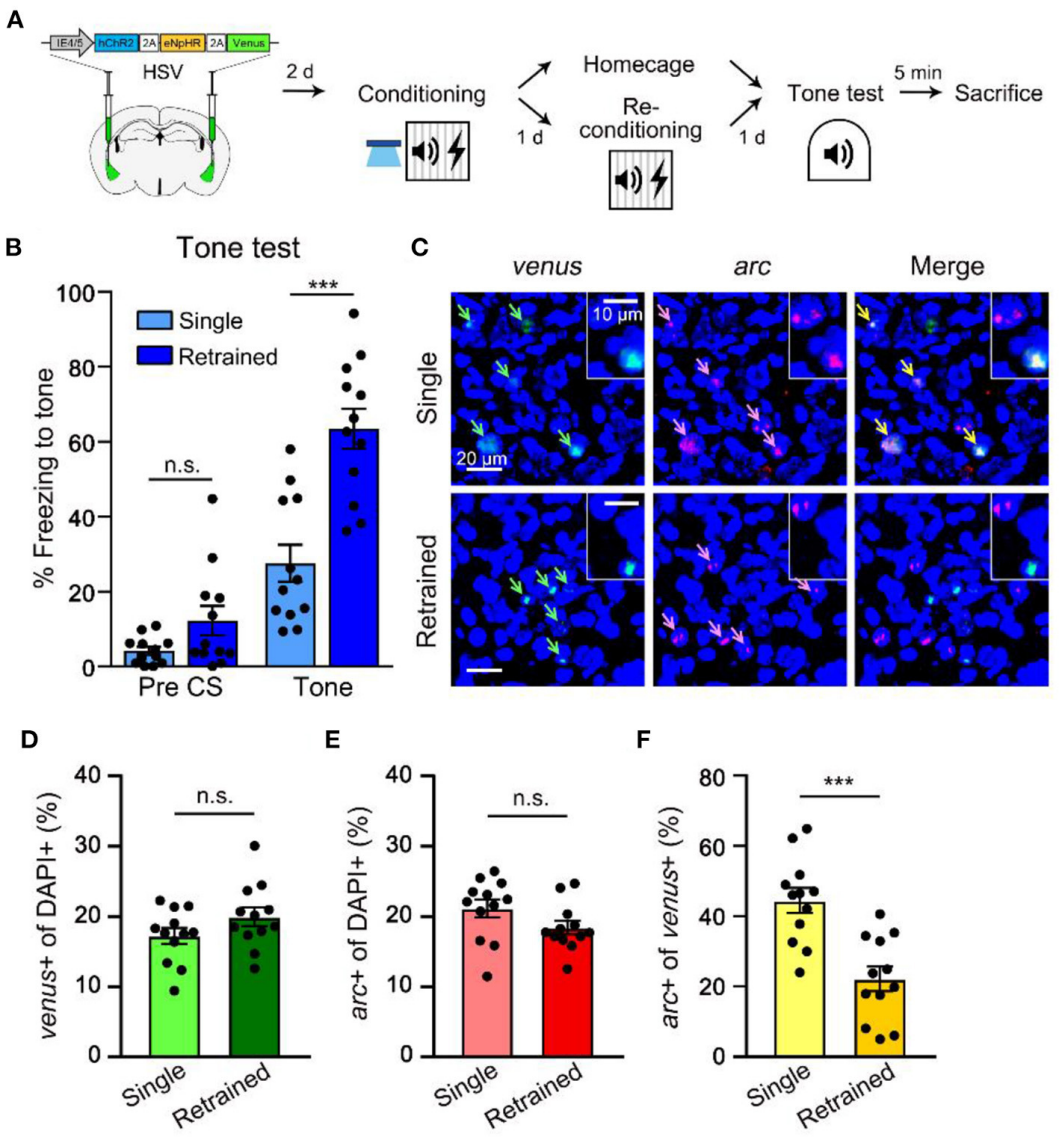
Retraining shifts a neuronal population activated during recall distinct from the originally allocated ensemble. **(A)** Schematic diagram of injection of HSV virus vector in the bilateral LA (left) and behavior scheme (right) for preparation of brain samples for FISH from single- and re-conditioned animals 5 min following memory recall. **(B)** Level of freezing measured from single (*n* = 12 mice) and re-conditioning (*n* = 12 mice) groups during tone test. **(C)** Representative confocal microscopic images of *venus* and *arc* RNA signals detected in the LA cells for single and re-conditioning groups. Scale bar, 20 μm; inset, 10 μm. **(D,E)** Proportion of *venus*+ cells **(C)** or the overall arc-induced cells **(D)** were comparable between the two groups. **(F)** Percentage of *arc*+ nuclei (induced by tone test) out of *venus*+ cells (excitability manipulated) in single vs. re-conditioning group. ***p < 0.001. n.s., not significant. Data are shown as mean ± SEM.

Next, we conducted imaging analysis to determine the change in neuronal ensembles supporting memory after retraining. Neural ensembles active during fear memory acquisition are preferentially reactivated during memory recall (Reijmers et al., 2007; Deng et al., 2013; Tayler et al., 2013; Tonegawa et al., 2015). To examine the reactivation probability of the initial engram during memory retrieval after retraining, we used *arc* FISH as a measure of recent neural activity (Guzowski et al., 1999; Han et al., 2007). We captured mRNA expression of *venus* to detect cells manipulated by optogenetic stimulation, and immediate early gene *arc* 5 min after memory recall to visualize cell population activated during memory recall with or without retraining (Figure 3A). Animals in the retraining group displayed significantly more freezing to tone compared to a single group, which indicates enhanced memory by retraining (Figure 3B). Cell counting of *venus*+ cells indicated a sparse viral infection consistent with our previous report (Kim et al., 2014). We first confirmed that *venus*+ LA cells displayed a significantly higher probability of overlap with *arc* signal if they were excited before conditioning (ChR2 ON) compared to the chance level probability measured in non-excited condition (ChR2 OFF; Supplementary Figure 4), validating preferential memory allocation to the optogenetically manipulated cells. In re-conditioning experiments, the overall size of *venus*- or *arc*-positive population was constant compared to the single training group (Figures 3C–E). However, the probability of *venus*+ cell population (initial memory-encoding cells) overlapping with *arc*+ cell population (induced by retrieval) was significantly lower in the 24 h retrained group compared to the single training group (Figures 3C,F). These results thus indicate that the initially allocated LA engram cells become less likely to be reactivated during memory recall after retraining, which was consistent with the optogenetic behavioral results. Since the size of *arc*+ population observed in the retraining group was similar to that of a single training group, our results suggest a shift of cell ensembles supporting memory by retraining.

### Initial Engram Cells Remain Stable If Retraining Occurs 6 h After Initial FC

**Figure 4:**
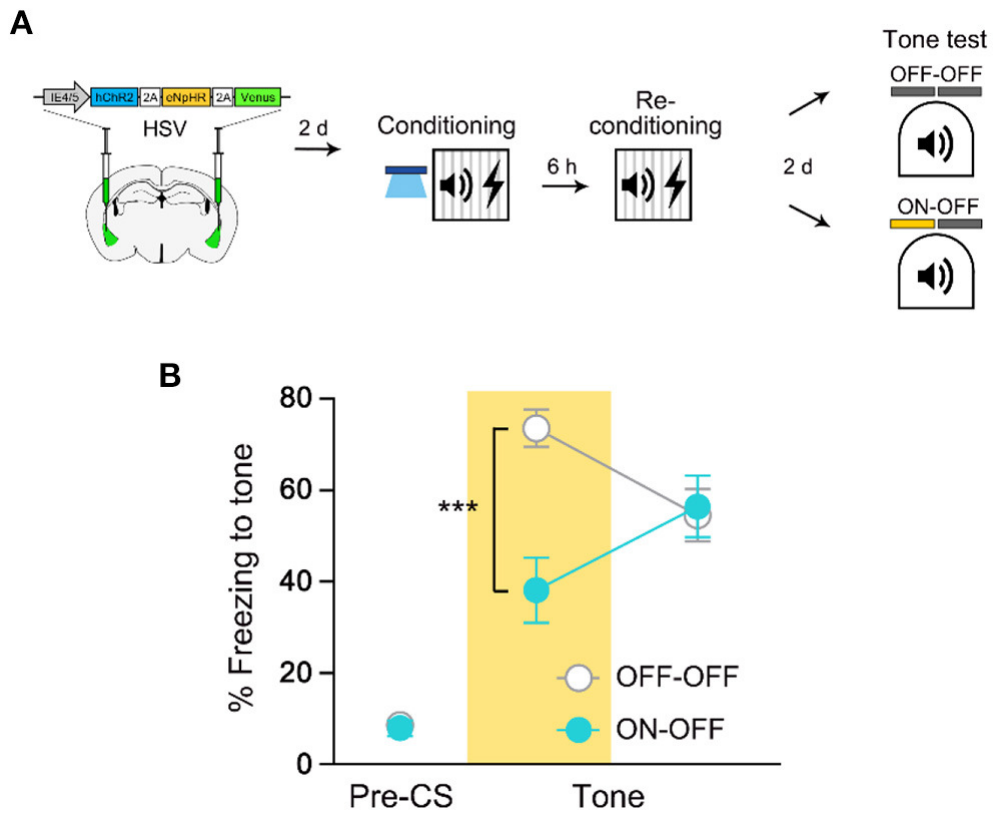
Re-conditioning in 6 h interval results in co-allocation of fear memory. **(A)** Schematic diagram of injection of HSV virus vector in the bilateral LA (left) and behavior experiment scheme (right). **(B)** Behavior results for tone recall test with (ON-OFF; *n* = 8 mice) or without (OFF-OFF; *n* = 7 mice) NpHR inhibition after 6 h re-conditioning. ***p < 0.001. Data are shown as mean ± SEM.

A previous study has shown that even two distinct FC events are encoded into an overlapping cell population if they occur close in time (Rashid et al., 2016). Hence, we asked whether retraining 6 h, instead of 24 h, after the initial FC similarly induces co-allocation of memory (Figure 4A). We performed NpHR experiment as described above but this time retraining occurred 6 h after the initial FC. We found that NpHR inhibition during the recall test significantly impaired freezing compared with a light-OFF control group (Figure 4B). This result indicates that as opposed to 24 h retraining, retraining with a shorter time interval after the initial training did not induce a shift in cell ensembles that encode the memory. Since we show significant inhibition of an equally strong memory (6 vs. 24 h retrained; Supplementary Figure 5) by NpHR inhibition, it is unlikely that the ineffectiveness of NpHR inhibition on 24 h retrained memory was due to the increased number of training trials or associative memory strength. These results thus further support that fear memory recall becomes independent of the initial fear engram after retraining in 24-h interval, regardless of memory strength.

### Optogenetic Excitation at the Time of Retraining Does Not Drive Memory Allocation

**Figure 5:**
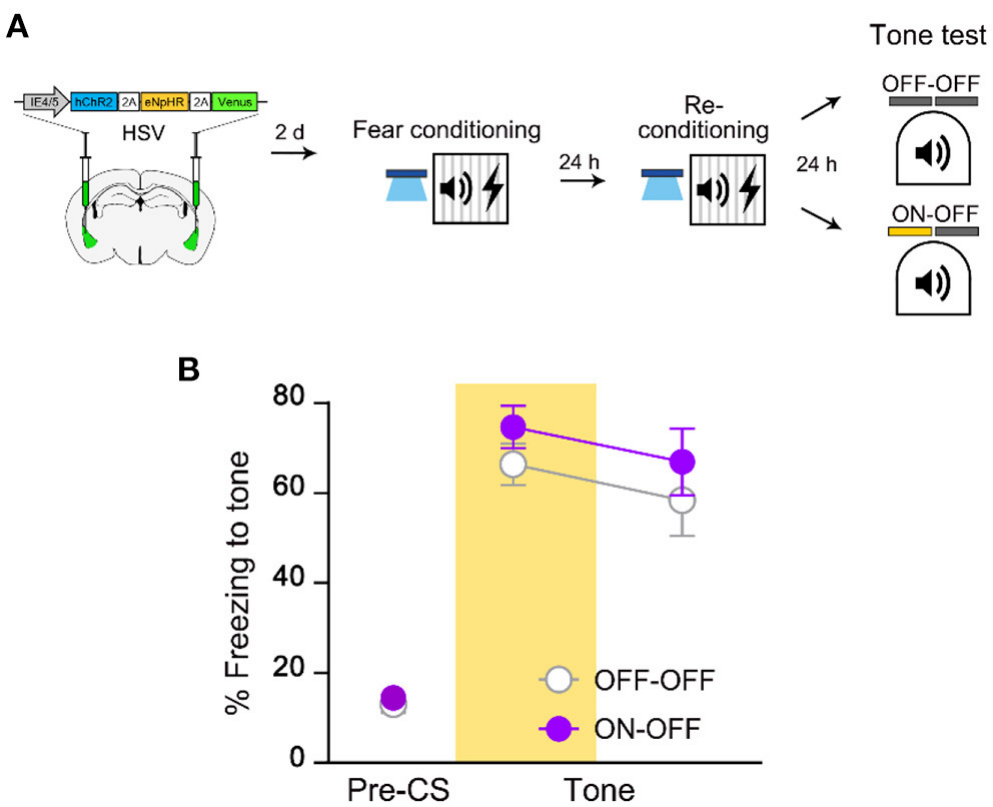
Retrained fear memory is no longer dependent on the initial memory-encoding LA ensemble despite increase in excitability. **(A)** Schematic diagram of injection of HSV virus vector in the bilateral LA (left) and behavior experiment scheme (right). **(B)** Behavior results for tone recall test with (ON-OFF; *n* = 8 mice) or without (OFF-OFF; *n* = 7 mice) NpHR inhibition after re-conditioning with increased excitability. Data are shown as mean ± SEM.

The results from 6-h retraining experiment led us to think that the excitability rule may also govern memory allocation of repeated events. To examine this possibility, we asked whether increasing excitability of the initial engram cells right before re-conditioning will allow retrained memory to be allocated into the same engram ensemble (Figure 5A). If excitability determines the fate of re-conditioned memory as for the initial allocation process, memory should be retained in the manipulated neurons even after retraining. However, we found that NpHR inhibition still had no effect on retrained memory recall even when the initial engram cells were re-excited prior to the 2nd conditioning (Figure 5B). We obtained the same results when excitability manipulation was presented only before re-conditioning (Supplementary Figure 6). By testing ChR2-driven allocation 3 days after HSV injection surgery and NpHR inhibition of fear memory the following day, we confirmed that both opsins were functional at their respective time points (Supplementary Figure 7). Together, these results suggest a novel mechanism other than excitability-dependent allocation rule that mediates reorganization in neuronal ensembles by retraining.

## Discussion

Using ArcCreER^T2^ transgenic mice to label neurons in the amygdala active during FC, we recently reported that cell ensembles are reorganized to support memory by retraining (Cho et al., 2021). Here, we used a different approach to target neurons allocated to the fear memory engram and essentially found the same result. When we increased excitability in a sparse subset of LA neurons at the time of training, we observed that these neurons were preferentially allocated into an engram, which confirms a previous report (Rashid et al., 2016). Our earliest results that show memory no longer depends on the initially recruited cells demonstrated that the original engram cells become unnecessary for memory recall by retraining. Moreover, retrieval of the fear memory after retraining activated a neural ensemble different from the initial ChR2-NpHR-expressing population that participated in encoding memory. It is unlikely that the retraining effect on neuronal ensembles was due to some non-specific long-term post-activation effect. If this is true, then the retraining protocol should produce the same effect on neuronal ensembles irrespective of time intervals (6 vs. 24 h) between training and retraining. However, this was not the case in this study. Moreover, recall alone instead of retraining did not induce the same changes in neuronal ensembles (Supplementary Figure 3). Furthermore, although it used a different cell labeling method, we previously found that shock alone is not effective either (Cho et al., 2021). These findings together strongly support that the retraining effect on neuronal ensembles is highly specific. These results are largely consistent with the finding we reported earlier, where synaptic disconnection in the original engram cells accompanied the shift in the memory engram following retraining (Cho et al., 2021). Thus, the current findings further strengthen the idea that fear memory is updated by forming a new engram with the inactivation of the original one in the LA after repetition of an associative training in 24-h interval.

Extending beyond the reports from Cho et al., we found a critical difference in the memory allocation process between initial training and retraining. Allocation for the first conditioning depended upon cellular excitability, as shown in Figure 1 and from other studies (Yiu et al., 2014; Cai et al., 2016; Rashid et al., 2016; Lisman et al., 2018), but retraining appeared to occur independent of the excitability rule, as demonstrated by our results in Figure 5 and Supplementary Figure 6. Rashid et al. reported the memories of two distinct auditory-conditioned stimuli (CS1 and CS2 with 24-h intertraining interval) that are normally dis-allocated to separate engrams can be co-allocated if excitability of the CS1 engram cells is elevated immediately before the CS2 event (Rashid et al., 2016). Such result highlights our finding where repeated training with the *same* CS resulted in allocation to distinct engrams even with the artificial manipulation of neuronal excitation. Excitability ruled for memory allocation for conditioning with distinct CSs, but not for re-conditioning with identical tone, which implies the presence of a novel allocation process that overrides the excitability-based competition. One limitation in this study, however, is that it is unknown which proportion of the infected cells were recruited each time they were activated (during training and retraining) and how much they overlap with each manipulation. A longitudinal recording of neuronal activity over-repeated learning using *in vivo* recording techniques such as Ca^2+^ imaging combined with simultaneous *in vivo* detection of HSV-infected neuronal population might be able to provide some answers in the future study. It should be noted that when retraining occurred 6 h after the initial FC, the same initially recruited cells kept participating in encoding the memory. These findings thus suggest that the novel process is specifically engaged by retraining occurring at the distant time point from the initial training. The engram changes by the retraining we observed are reminiscent of the idea that just as the memories for events occurring close in time are linked, and memories for events occurring distant in time may need to be separated (Cai et al., 2016; Eichenbaum, 2016; Rashid et al., 2016). Therefore, our findings may provide an explanation to how the brain specifically distinguishes memories for similar events experienced at different time points. Such memory separation should also be critical for properly linking the same, but temporally separated, events.

It has been suggested that parvalbumin interneuron-mediated inhibitory network is specifically engaged for the allocation of two distinct memories in the LA if they occur close in time and restricts memory allocation to overlapping cell populations by suppressing non-allocated neurons (Rashid et al., 2016). Given our data from 6-h experiment, we assume that perhaps, similar inhibitory mechanism is also involved in regulating memory allocation for repeated events with 6-h interval. Nevertheless, it is unclear whether the LA neurons allocated into an engram by optogenetic excitation show increased excitability lasting 6 h after training. Using electrophysiological approaches, a previous study in the dentate gyrus has shown that in response to the natural recall process, engram cells only transiently increase their excitability no more than 3 h (Pignatelli et al., 2019). Therefore, to clarify this issue, the membrane properties such as resting membrane potential, action potential threshold, rheobase, and membrane resistance need to be determined in the LA engram neurons and their neighbors at different time points after training in the future investigation.

It is currently unclear what mechanisms underlie memory allocation for a repeated event. Previously, we found that the dendritic spine density of initial LA engram cells is downregulated by retraining 24 h following initial training (Cho et al., 2021). This synaptic change could be a mechanism leading to the inactivation of initial engram cells. Given the lack of effect of excitability manipulation at the time of retraining on memory allocation, it is unlikely that such spine inactivation is triggered by decreased excitability. Because reorganization in cell ensembles was effective only at the presence of both tone and shock, but not each stimulus alone (this study and Cho et al., 2021), we consider that a specific input activity pattern that requires inputs from both tone (same tone) and shock is critical for inducing the reorganization process in the engram cell populations. In this regard, it needs to be tested in the future study whether activation of the pre-existing engram cells *via* specific input pathways is critical for the turnover of cell ensembles by retraining. Our study suggests that reorganization of engram by repeated experience is not a stochastic process but is rather determined by a novel process that probably depends on pre-existing pattern of the engram.

The formation of a new distinct engram with inactivation of the old engram cells adds a new dimension to brain mechanisms of memory update. Understanding this process will explain how associative memory persists over time through repeated experience and help to develop treatments for patients who suffer from chronic repeated trauma.

## Data Availability Statement

The original contributions presented in the study are included in the article/Supplementary Material, further inquiries can be directed to the corresponding author/s.

## Ethics Statement

The animal study was reviewed and approved by the KAIST Institutional Animal Care and Use Committee.

## Author Contributions

H-YC and J-HH conceived the idea, designed all of the experiments, analyzed the data, discussed the results, and wrote the manuscript with inputs from all authors. H-YC performed the virus surgery, animal behavior experiments, FISH, cell counting analysis, immunohistochemistry, and imaging. H-SL assisted behavior experiments. YJ performed the *in vivo* optrode recording and data analysis. JH assisted the FISH. MY performed the western blot assay. J-HH directed the study. All authors contributed to the article and approved the submitted version.

## Funding

This work was supported by the grants from Samsung Science and Technology Foundation (project number SSTF-BA1801-10 to J-HH). The funder was not involved in the study design, collection, analysis, interpretation of data, the writing of this article or the decision to submit it for publication.

## Conflict of Interest

The authors declare that the research was conducted in the absence of any commercial or financial relationships that could be construed as a potential conflict of interest.

## Publisher's Note

All claims expressed in this article are solely those of the authors and do not necessarily represent those of their affiliated organizations, or those of the publisher, the editors and the reviewers. Any product that may be evaluated in this article, or claim that may be made by its manufacturer, is not guaranteed or endorsed by the publisher.
