# Supplementary material for: Excitability-Independent Memory Allocation for Repeated Event
Source: Front Behav Neurosci. 2022 Apr 27;16:860027. doi: 10.3389/fnbeh.2022.860027 (PMC9094695; doi:10.3389/fnbeh.2022.860027)
Supplement: Supplementary file 1 [file Data_Sheet_1.docx]

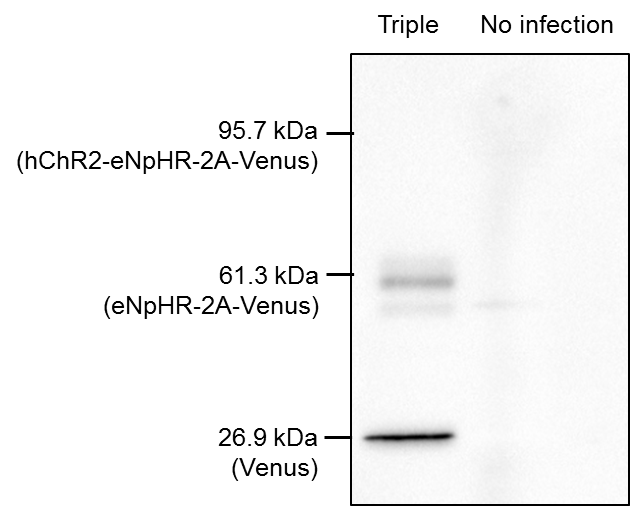


Supplementary Figure 1. Efficient cleavage of hChR2, eNpHR and Venus by the 2A peptide. Related to Figure 1

Western blot of protein extracts from HSV-hChR2-2A-eNpHR-2A-Venus infected HEK293T cultured cells. In HSV infection condition, un-cleaved full-length protein (95.7 kDa) was undetectable but there was strong expression of Venus protein (26.9 kDa) and relatively weak expression of residual eNpHR-2A-Venus fusion protein (61.3 kDa). In no infection condition, no signal was detected.


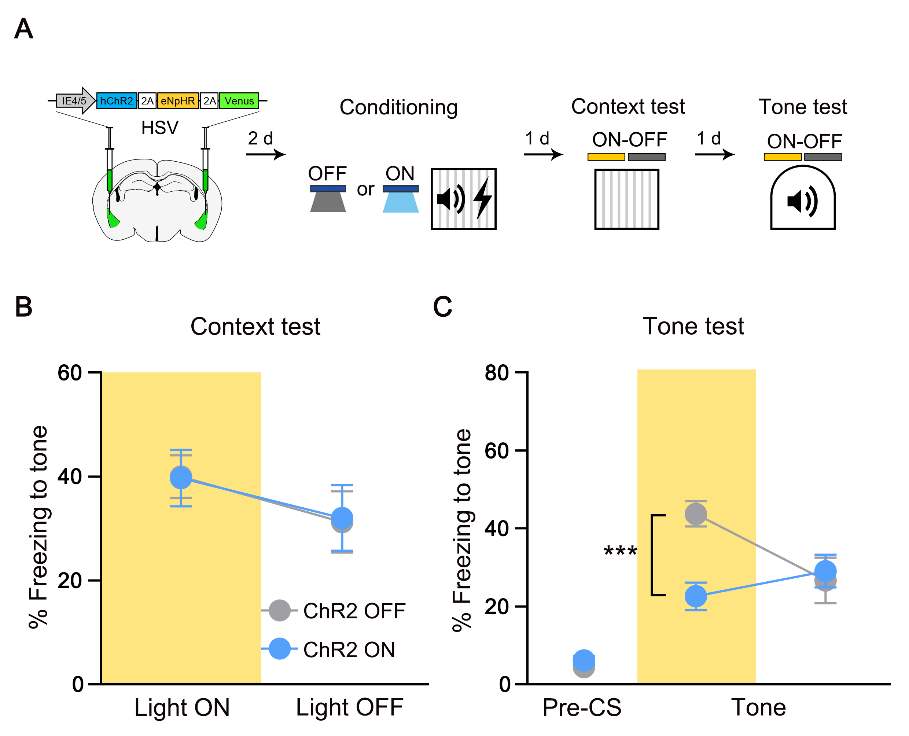


**Supplementary Figure 2. Optogenetic inhibition of HSV-infected neurons have no effect on context memory recall. Related to Figure 1**

(A) Schematic diagram of injection of HSV-hChR2-2A-eNpHR-2A-Venus virus vector in the bilateral LA (left) and the behavior scheme for testing memory recall for context and tone (right).

(B and C) Freezing levels measured for ChR2 OFF (*n* = 8) and ChR2 ON (*n* = 9) groups during context (B) and tone (C) memory recall test. Yellow shading indicates 561 nm light illumination to activate NpHR.

****P* < 0.001. Data are shown as mean ± s.e.m.


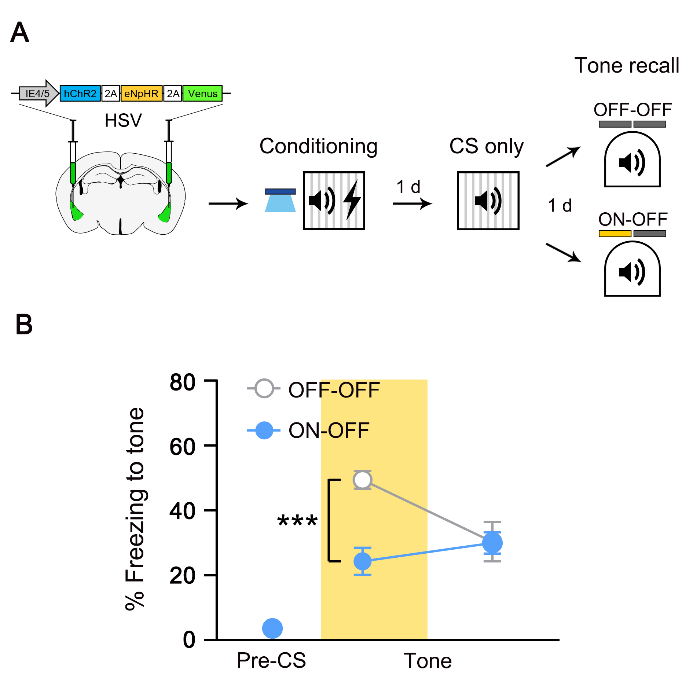


Supplementary Figure 3. Recall of fear memory after exposure to CS depends on initially-allocated engram cells. Related to Figure 2

(A) Schematic diagram of injection of HSV-hChR2-2A-eNpHR-2A-Venus virus vector in the bilateral LA (left) and the behavior scheme for exposing the animals to CS 24 h after fear conditioning to the tone (right).

(B) Freezing levels measured for NpHR ON-OFF (*n* = 10) and NpHR OFF-OFF (*n* = 9) groups during tone memory recall test. Yellow shading indicates 561 nm light illumination to activate NpHR.

****P* < 0.001. Data are shown as mean ± s.e.m.


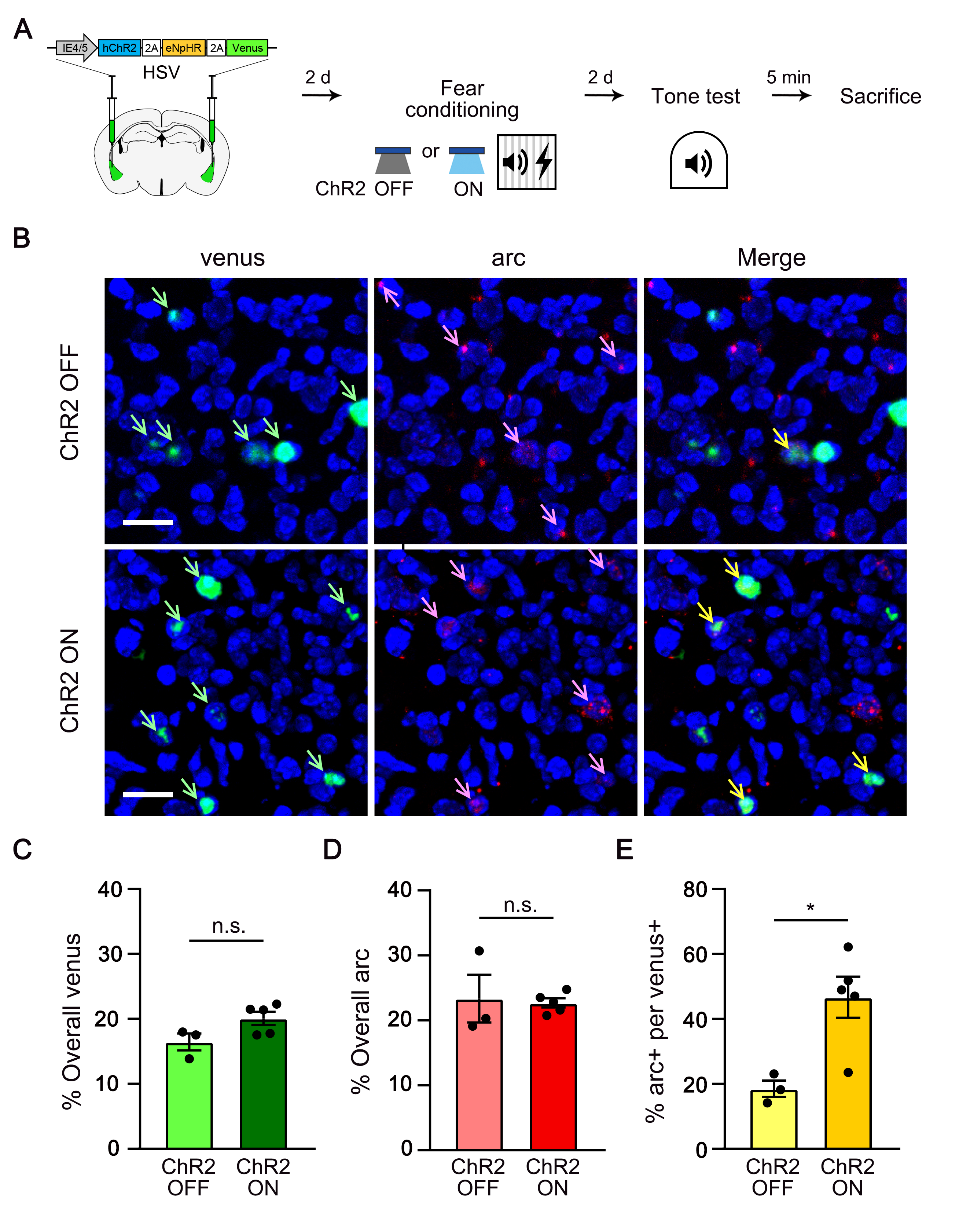


Supplementary Figure 4. Arc induction is only detected in cells that were more excitable during conditioning. Related to Figure 3

(A) Schematic diagram of injection of HSV virus vector in the bilateral LA (left) and behavior experiment scheme (right). One group received ChR2 stimulation immediately before auditory fear conditioning (ChR2 ON), while the other group did not (ChR2 OFF). Animals were sacrificed 5 min after tone test.

(B) Representative confocal microscopic images of *venus* and *arc* RNA signals detected in the LA cells for ChR2 OFF and ChR2 ON groups. Scale bar, 20 μm.

(C and D) Proportion of *venus*+ cells (C) or the overall arc-induced cells (D) were comparable between the two groups (ChR2 OFF *n* = 3, ChR2 ON *n* = 5).

(E) Overlap percentage of *arc*+ nuclei out of *venus*+ cells was significantly higher in ChR2 ON group.

**P* < 0.05. n.s., not significant. Data are shown as mean ± s.e.m.


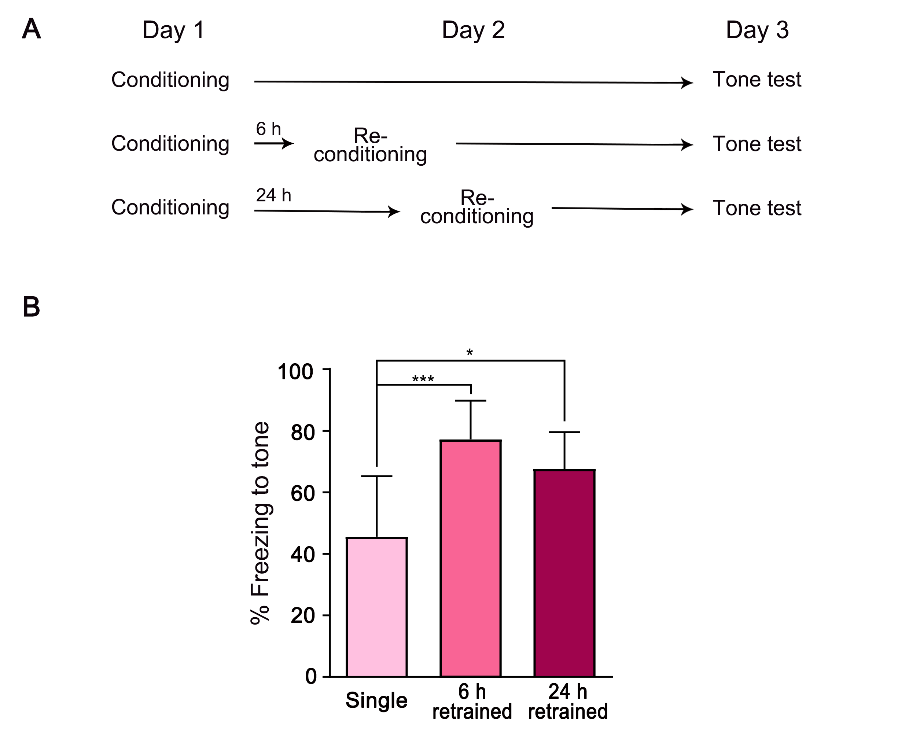


**Supplementary Figure 5. Freezing levels measured for single trained, 6 h retrained and 24 h retrained naïve animals. Related to Figure 4**

(A) Behavior scheme to test freezing levels in single trained and retrained (6 h and 24 h intervals) groups.

(B) Retrained groups (*n* = 10 for 6 h; *n* = 8 for 24 h) displayed higher freezing levels compared to single trained animals (*n* = 10).

**P* < 0.05, ****P* < 0.001. Data are shown as mean ± s.e.m.


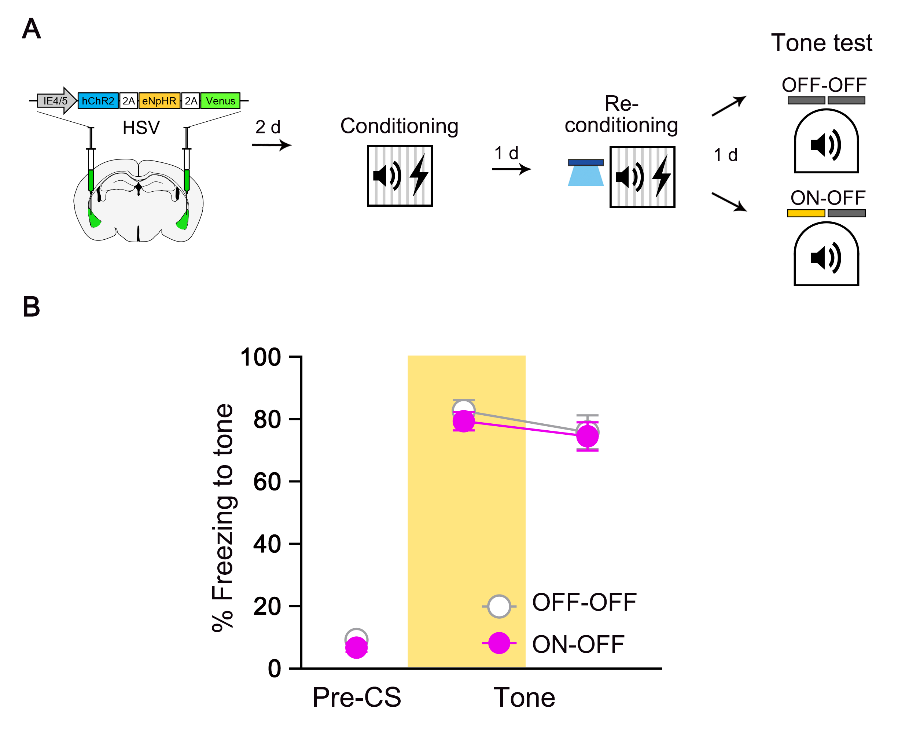


**Supplementary Figure 6. Increasing excitability immediately before re-conditioning does not allocate memory to the manipulated neurons. Related to Figure 5**

(A) Schematic diagram of injection of HSV-hChR2-2A-eNpHR-2A-Venus virus vector in the bilateral LA (left) and the behavior scheme for increasing excitability immediately before re-conditioning (right).

(B) Behavior results for tone recall test with (ON-OFF; *n* = 8) or without (OFF-OFF; *n* = 8) NpHR inhibition after increasing excitability only before re-conditioning.

Data are shown as mean ± s.e.m.


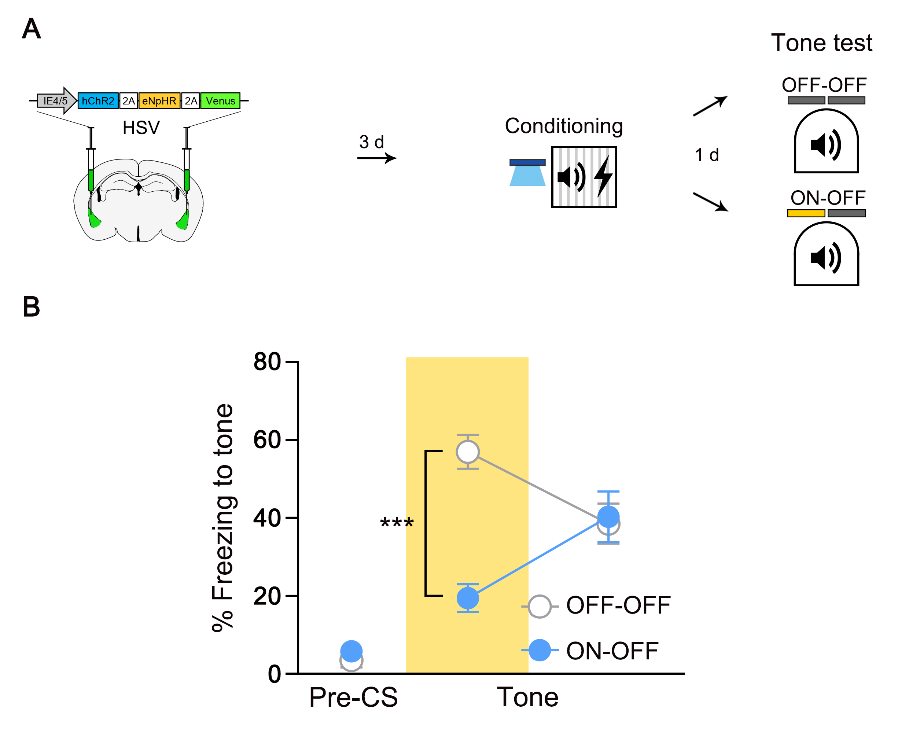


**Supplementary Figure 7. ChR2 and NpHR opsins are still functional 3 days after virus infection. Related to Figure 5**

(A) Schematic diagram of injection of HSV-hChR2-2A-eNpHR-2A-Venus virus vector in the bilateral LA (left) and the behavior scheme conducted 3 days later (right).

(B) Behavior results for tone recall test with (ON-OFF; *n* = 5) or without (OFF-OFF; *n* = 5) NpHR inhibition in mice that received excitability manipulation 3 days after virus injection.

****P* < 0.001. Data are shown as mean ± s.e.m.

Supplementary Table 1. Statistical analysis details related to Figures 1-5.

| **Figure** | **Group** | **Measurement variable** | **Statistical analysis** | **Effects of ANOVA** | **Statistical value** | ***P* value** | **Significance** |
| --- | --- | --- | --- | --- | --- | --- | --- |
| Fig. 1D | ChR2 OFF  (*n* = 10) | % Freezing | 2-way RM ANOVA | Group (ChR2 OFF vs ChR2 ON) | *F*_1, 21_ = 7.744 | 0.0112 | * |
|  | ChR2 ON  (*n* = 13) |  |  | Stimulus (Pre CS vs Tone+NpHR vs Tone) | *F*_2, 42_ = 110.6 | < 0.0001 | *** |
|  |  |  |  | Group × Stimulus | *F*_2, 42_ = 27.29 | < 0.0001 | *** |
|  |  |  | Sidak's multiple comparison | Pre CS |  | 0.9760 |  |
|  |  |  |  | NpHR ON |  | < 0.0001 | *** |
|  |  |  |  | NpHR OFF |  | 0.9678 |  |
| Fig. 2B | OFF-OFF  (*n* = 7) | % Freezing | 2-way RM ANOVA | Group (OFF-OFF vs ON-OFF) | *F*_1,13_ = 0.1316 | 0.7226 |  |
|  | ON-OFF  (*n* = 8) |  |  | Stimulus (Pre CS vs Tone+NpHR vs Tone) | *F*_2, 26_ = 118 | <0.0001 | *** |
|  |  |  |  | Group × Stimulus | *F_2_*_, 26_ = 1.006 | 0.3796 |  |
|  |  |  | Sidak's multiple comparison | Pre CS |  | 0.9998 |  |
|  |  |  |  | NpHR OFF or ON |  | 0.9962 |  |
|  |  |  |  | NpHR OFF |  | 0.5963 |  |
| Fig. 2C | OFF-OFF  (*n* = 9) | % Freezing | 2-way RM ANOVA | Group (OFF-OFF vs ON-OFF) | *F*_1,16_ = 5.273 | 0.0355 | * |
|  | ON-OFF  (*n* = 9) |  |  | Stimulus (Pre CS vs Tone+NpHR vs Tone) | *F*_2, 32_ = 57.91 | < 0.0001 | *** |
|  |  |  |  | Group × Stimulus | *F*_2, 32_ = 7.782 | 0.0018 | ** |
|  |  |  | Sidak's multiple comparison | Pre CS |  | 0.9952 |  |
|  |  |  |  | NpHR OFF or ON |  | 0.0001 | *** |
|  |  |  |  | NpHR OFF |  | 0.9977 |  |
| Fig. 3B | Single  (*n* = 12) | % Freezing | 2-way RM ANOVA | Group (Single vs Retrain) | *F*_1,11_ = 16.73 | 0.0018 | ** |
|  | Retrained (*n* = 12) |  |  | Stimulus (Pre CS vs Tone) | *F*_1,11_ = 295.8 | <0.0001 | *** |
|  |  |  |  | Group × Stimulus | *F*_1,11_ = 10.36 | 0.0082 | ** |
|  |  |  | Sidak's multiple comparison | Pre CS |  | 0.3900 |  |
|  |  |  |  | Tone |  | 0.0002 | *** |
| Fig. 3D | Single  (*n* = 12) | % *venus* population | unpaired t-test |  | *t* = 1.565, df = 22 | 0.1318 |  |
|  | Retrained (*n* = 12) |  |  |  |  |  |  |
| Fig. 3E | Single  (*n* = 12) | % *arc* population | unpaired t-test |  | *t* = 1.723, df = 22 | 0.0989 |  |
|  | Retrained (*n* = 12) |  |  |  |  |  |  |
| Fig. 3F | Single  (*n* = 12) | % Overlap | unpaired t-test |  | *t* = 4.507, df = 22 | 0.0002 | *** |
|  | Retrained (*n* = 12) |  |  |  |  |  |  |
| Fig. 4B | OFF-OFF (*n* = 7) | % Freezing | 2-way RM ANOVA | Group (OFF-OFF vs ON-OFF) | *F*_1,13_ = 3.705 | 0.0764 |  |
|  | ON-OFF  (*n* = 8) |  |  | Stimulus (Pre CS vs Tone+NpHR vs Tone) | *F*_2, 26_ = 107.6 | < 0.0001 | *** |
|  |  |  |  | Group × Stimulus | *F*_2, 26_ = 15.64 | < 0.0001 | *** |
|  |  |  | Sidak's multiple comparison | Pre CS |  | 0.9996 |  |
|  |  |  |  | NpHR OFF or ON |  | < 0.0001 | *** |
|  |  |  |  | NpHR OFF |  | 0.9911 |  |
| Fig. 5B | OFF-OFF (*n* = 7) | % Freezing | 2-way RM ANOVA | Group (OFF-OFF vs ON-OFF) | *F*_1,19_ = 0.9394 | 0.3446 |  |
|  | ON-OFF (*n* = 8) |  |  | Stimulus (Pre CS vs Tone+NpHR vs Tone) | *F*_2, 38_ = 133.1 | < 0.0001 | *** |
|  |  |  |  | Group × Stimulus | *F*_2, 38_ = 0.5719 | 0.5692 |  |
|  |  |  | Sidak's multiple comparison | Pre CS |  | 0.9970 |  |
|  |  |  |  | NpHR OFF or ON |  | 0.6326 |  |
|  |  |  |  | NpHR OFF |  | 0.6075 |  |

Supplementary Table 2. Statistical analysis details related to Supplementary Figures 1-7.

| **Figure** | **Group** | **Measurement variable** | **Statistical analysis** | **Effects of ANOVA** | **Statistical value** | ***P* value** | **Significance** |
| --- | --- | --- | --- | --- | --- | --- | --- |
| Fig. S2B | ChR2 OFF (*n* = 8) | % Freezing | 2-way RM ANOVA | Group (ChR2 OFF vs. ChR2 ON) | *F*_1, 15_ = 0.0011 | 0.9732 |  |
|  | ChR2 ON (*n* = 9) |  |  | Stimulus (NpHR ON vs. NpHR OFF) | *F*_1, 15_ = 10.8 | 0.0050 | ** |
|  |  |  |  | Group × Stimulus | *F*_1, 15_ = 0.0453 | 0.8342 |  |
|  |  |  | Sidak's multiple comparison | NpHR ON |  | 0.9992 |  |
|  |  |  |  | NpHR OFF |  | 0.9938 |  |
| Fig. S2C | ChR2 OFF (*n* = 8) | % Freezing | 2-way RM ANOVA | Group (ChR2 OFF vs. ChR2 ON) | *F*_1, 15_ = 3.035 | 0.1019 |  |
|  | ChR2 ON (*n* = 9) |  |  | Stimulus (Pre CS vs Tone+NpHR vs Tone) | *F_2_*_, 30_ = 40.1 | < 0.0001 | *** |
|  |  |  |  | Group × Stimulus | *F_2_*_, 30_ = 8.258 | 0.0014 | ** |
|  |  |  | Sidak's multiple comparison | Pre CS |  | 0.9808 |  |
|  |  |  |  | NpHR ON |  | 0.0004 | *** |
|  |  |  |  | NpHR OFF |  | 0.9507 |  |
| Fig. S3B | OFF-OFF (*n* = 9) | % Freezing | 2-way RM ANOVA | Group (OFF-OFF vs ON-OFF) | *F*_1, 17_ = 4.988 | 0.0393 | * |
|  | ON-OFF  (*n* = 10) |  |  | Stimulus (Pre CS vs Tone+NpHR vs Tone) | *F*_2, 34_ = 79.89 | < 0.0001 | *** |
|  |  |  |  | Group × Stimulus | *F*_2, 34_ = 13.41 | < 0.0001 | *** |
|  |  |  | Sidak's multiple comparison | Pre CS |  | > 0.9999 |  |
|  |  |  |  | NpHR OFF or ON |  | < 0.0001 | *** |
|  |  |  |  | NpHR OFF |  | 0.9998 |  |
| Fig. S4C | ChR2 OFF (*n* = 3) | % *venus* population | unpaired t-test |  | *t* = 2.178, df = 6 | 0.0723 |  |
|  | ChR2 ON (*n* = 5) |  |  |  |  |  |  |
| Fig. S4D | ChR2 OFF (*n* = 3) | % *arc* population | unpaired t-test |  | *t* = 0.2322, df = 6 | 0.8241 |  |
|  | ChR2 ON (*n* = 5) |  |  |  |  |  |  |
| Fig. S4E | ChR2 OFF (*n* = 3) | % Overlap | unpaired t-test |  | *t* = 3.259, df = 6 | 0.0173 | * |
|  | ChR2 ON (*n* = 5) |  |  |  |  |  |  |
| Fig. S5B | Single  (*n* = 8) | % Freezing | 1-way ANOVA |  | *F*_2, 24_ = 10.53 | 0.0005 | *** |
|  | 6 h retrained (*n* = 10) |  | Tukey's multiple comparison | Single vs. 6h retrain |  | 0.0004 | *** |
|  | 24 h retrained  (*n* = 8) |  |  | Single vs. 24h retrain |  | 0.0246 | * |
|  |  |  |  | 6h retrain vs. 24h retrain |  | 0.2949 |  |
| Fig. S6B | OFF-OFF (*n* = 8) | % Freezing | 2-way RM ANOVA | Group (OFF-OFF vs ON-OFF) | *F*_1, 14_ = 0.319 | 0.5812 |  |
|  | ON-OFF  (*n* = 8) |  |  | Stimulus (Pre CS vs Tone+NpHR vs Tone) | *F*_2, 28_ = 532.4 | < 0.0001 | *** |
|  |  |  |  | Group × Stimulus | *F*_2, 28_ = 0.0717 | 0.9310 |  |
|  |  |  | Sidak's multiple comparison | Pre CS |  | 0.9507 |  |
|  |  |  |  | NpHR OFF or ON |  | 0.8920 |  |
|  |  |  |  | NpHR OFF |  | 0.9904 |  |
| Fig. S7B | OFF-OFF (*n* = 5) | % Freezing | 2-way RM ANOVA | Group (OFF-OFF vs ON-OFF) | *F*_1, 8_ = 5.585 | 0.0457 |  |
|  | ON-OFF  (*n* = 5) |  |  | Stimulus (Pre CS vs Tone+NpHR vs Tone) | *F*_2, 16_ = 77.41 | < 0.0001 | *** |
|  |  |  |  | Group × Stimulus | *F*_2, 16_ = 25.89 | < 0.0001 | *** |
|  |  |  | Sidak's multiple comparison | Pre CS |  | 0.9759 |  |
|  |  |  |  | NpHR OFF or ON |  | < 0.0001 | *** |
|  |  |  |  | NpHR OFF |  | 0.9884 |  |
